# Redirecting Alzheimer's disease therapeutics: Multitarget drugs and complementary non‐pharmacological strategies

**DOI:** 10.1002/alz.71589

**Published:** 2026-06-23

**Authors:** Humberto Martínez‐Orozco, Jesús Andrade‐Guerrero, Nelly Jovana Pastén‐Castrejón, Chryslaine Rodríguez‐Tanty, Sofía Y Diaz‐Miranda

**Affiliations:** ^1^ Institute of Neurobiology Campus Juriquilla National Autonomous University of Mexico Queretaro Mexico; ^2^ Department of Neurochemistry Cuban Center for Neurosciences Havana Cuba

**Keywords:** mixed therapies, multifactorial disease, multifunctional drugs, neurodegenerative disorder, pharmacological therapies, SOPHI‐AD

## Abstract

Alzheimer's disease (AD) is a multifactorial neurodegenerative disorder driven by intersecting pathological processes. Persistent attrition in AD drug‐development pipelines highlights the limited clinical impact of single‐target therapies and has increased interest in multi‐target approaches acting on shared biological hubs. Although recent strategies extend beyond amyloid‐centered interventions, much of the evidence supporting multifunctional compounds remains preclinical or shows heterogeneous outcomes in advanced clinical stages. This narrative review examines drug candidates that have entered Phase III clinical trials in the last decade, with emphasis on emerging multitarget pharmacotherapies such as muscarinic M1 receptor agonists, dual M1/sigma‐1 receptor (σ1R) agonists, and dual σ1R agonist/anti‐amyloid agents. We also discuss complementary non‐pharmacological interventions, including physical exercise and targeted nutrition, that may support cognitive and emotional outcomes. Finally, we propose the Synergistic Optimized Pharmacological and Holistic Interventions for AD (SOPHI‐AD) framework as a conceptual and testable approach integrating multitarget pharmacology with lifestyle‐based strategies for AD management.

## Highlights

- Alzheimer's disease is a multifactorial network disorder making single‐pathway therapies intrinsically limited.
- Clinical pipelines show higher Phase III failure rate, historical dominance and shortcomings of amyloid‐centric strategies, and the need for multitarget approaches.
- Emerging multitarget pharmacotherapies—such as M1 muscarinic agonists, dual M1/σ1R agonists, and σ1R agonist/anti‐amyloid agent—demonstrate convergent neuroprotective actions.
- Non‐pharmacological interventions exert complementary multi‐mechanistic effects.
- Integration of multi‐target drugs with structured lifestyle and device‐based interventions aim to maximize mechanistic convergence, slow disease progression, and preserve functional independence and quality of life.

## 1 INTRODUCTION

Alzheimer's disease (AD) accounts for ≈70% of dementia cases worldwide and remains a leading cause of disability and dependency in older adults.^1^ The epidemiology of AD has raised serious concern among governments and health organizations due to its growing economic burden. There is currently no cure or fully effective treatment against AD. With annual costs exceeding one trillion dollars, AD is a major public health priority in aging populations.^2^ Disease progression severely impairs quality of life: early mild memory loss evolves into disorientation, mood and behavioral changes, attentional deficits, visuospatial and executive dysfunction, and sometimes delusions and hallucinations.^3^ Advancing stages produce marked disability, loss of independence, and the need for specialized care. Accordingly, effective therapies should aim to not only delay progression through early diagnosis but also repair the existing pathology to some extent.

AD etiology is multifactorial and can be manifested sporadically or genetically. Sporadic AD represents >95% of cases and remains of largely unknown etiology, whereas familial AD accounts for 1%–5%.^4^ Two hallmark processes are consistently implicated: first, the accumulation of amyloid beta (Aβ)—a peptide derived from amyloid precursor protein (APP)—forming neuritic plaques; second, the accumulation of hyperphosphorylated tau, forming neurofibrillary tangles. These lesions disrupt neuronal structure and function, drive neuronal death, and culminate in brain atrophy.^5^ In parallel, additional processes—including neuroinflammation, oxidative stress, microbiota dysbiosis, blood–brain barrier dysfunction, and vascular and synaptic alterations—contribute to disease onset and progression.^6^

Given the complexity of AD there are three central issues: prevention, early diagnosis, and treatment.^7^ Global prevention and early‐detection initiatives highlight the importance of modifiable risk factors, yet therapeutic options remain limited. On the other hand, diagnostic challenges continue to limit progress. Although definitive diagnosis still requires post‐mortem histopathology, combinations of clinical assessment with blood, cerebrospinal fluid (CSF), and neuroimaging biomarkers can reach ≈85%–90% accuracy. Nonetheless, more specific and accessible biomarkers are needed to enable even earlier detection.^7^, ^8^, ^9^ Therapeutic options remain restricted, and drug classes approved by the U.S. Federal Drug Administration (FDA) include (1) acetylcholinesterase inhibitors (donepezil, rivastigmine, galantamine), (2) the *N*‐methyl‐d‐aspartate (NMDA) receptor antagonist memantine, and (3) anti‐Aβ monoclonal antibodies (mAb; in example, aducanumab, donanemab). Unfortunately, these treatments show only modest effects on disease progression and clinical outcomes.^10^, ^11^ Their limited efficacy likely stems from targeting single pathways in what is fundamentally a multifactorial disorder with numerous interacting pathophysiological mechanisms. This limitation has sparked growing interest in multi‐target strategies that simultaneously address various pathological processes, potentially improving therapeutic efficacy and providing a more comprehensive approach to AD treatment.^12^

The present narrative review critically examines potential multi‐target pharmacological and non‐pharmacological strategies in AD, with four specific aims: (1) to assess mechanistic rationale and evidentiary limitations of selected multitarget approaches; (2) to analyze representative examples of multifunctional compounds; (3) to highlight translational barriers; and (4) to introduce the Synergistic Optimized Pharmacological and Holistic Interventions for AD (SOPHI‐AD) framework as a conceptual proposal for future strategies.

## 2 RETHINKING THERAPEUTIC APPROACHES FOR A MULTIFACTORIAL DISEASE

AD therapeutics has progressed through multiple cycles of optimism and disappointment, reflecting the complexity of translating mechanistic insight into clinical benefit. Longitudinal analyses of clinical pipelines have documented persistently high attrition of 99.6% from 2002–2012.^13^ During this period, only memantine (NMDA receptor antagonist) received approval for marketing by the FDA in 2004, indicating that target engagement alone may not guarantee meaningful cognitive or functional outcomes.

Amyloid‐centric approaches dominated late‐stage development for years. This hypothesis sustains that Aβ aggregation is the primary contributor to the disease, triggering alterations in signaling pathways, including tau phosphorylation and kinase‐mediated enzyme activation, and subsequently increasing oxidative stress, promoting mitochondrial and lysosomal dysfunction, and actively stimulating the inflammatory response.^14^ Such alterations, combined with the accumulation of Aβ oligomeric species, lead to neurodegeneration that impairs synaptic plasticity mechanisms, such as neurotransmission and neurogenesis—processes crucial for cognitive functions.^14^ However, AD etiology also involves Aβ‐independent mechanisms.^15^ Thus, repeated clinical late‐stage failures and mixed clinical signals have driven a broader exploration of tau‐directed, neuroinflammatory, synaptic, metabolic, and neuroprotective mechanisms.

Rather than framing these outcomes as a simple “failure of amyloid,” a more informative interpretation is that AD biology and trial populations appears to be heterogeneous. As a consequence, international organizations and governments have prioritized AD, launching initiatives to accelerate drug development by 2025.^16^, ^17^ However, treatment effects may depend on disease stage, patient stratification (including genetic and biomarker‐defined subgroups^18^), and the alignment of endpoints with mechanisms. Pipeline monitoring has also shown that non‐efficacy discontinuations frequently reflect issues of tolerability, dosing constraints, operational feasibility, or strategic company decisions, each with different implications for scientific inference, which is not limited only to AD drug candidates focused on Aβ.^19^, ^20^, ^21^, ^22^, ^23^, ^24^, ^25^, ^26^, ^27^, ^28^

**FIGURE 1:**
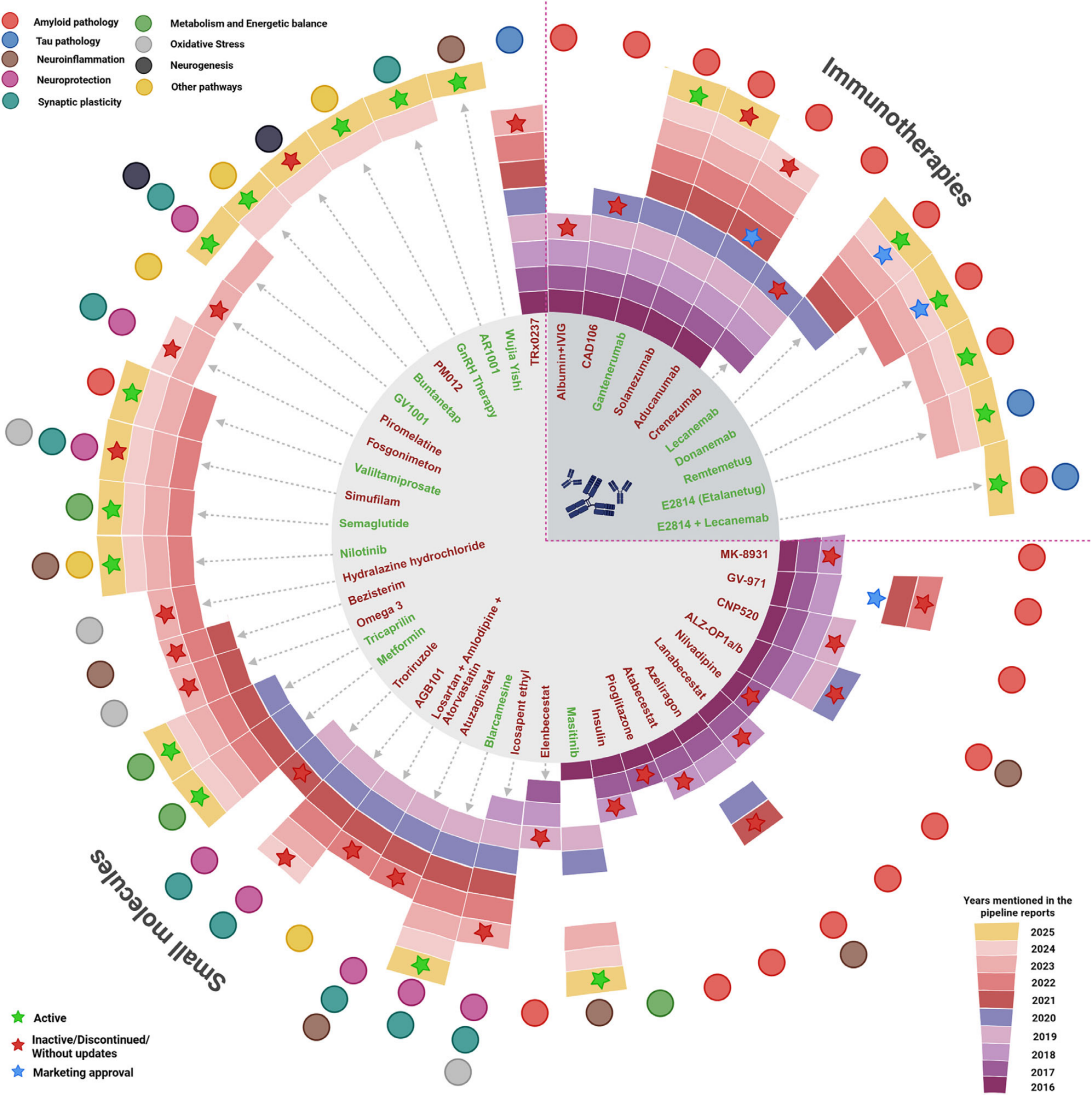
Timeline of therapies in Phase III clinical trials from 2016 and 2025. The central grey circle groups active (green words) and inactive (red words) therapies into immunotherapies (n = 11) and small molecules (n = 36). Each agent's Phase III timeline (n = 47) is shown with a color gradient. Mechanisms of action are indicated by colored circles (amyloid, tau, neuroinflammation, neuroprotection, synaptic plasticity, metabolism/energetics, oxidative stress, neurogenesis, others). Current trial status uses stars: red = inactive (adverse events, lack of efficacy, no updates, or company decisions), green = active, blue = year of marketing approval. This figure was elaborated using BioRender.

To better understand trends in the targets of AD drug candidates at advanced clinical stages, in the present narrative literature review, we have revised and subtracted information from pipelines published by Cummings and colleagues.^19^, ^20^, ^21^, ^22^, ^23^, ^24^, ^25^, ^26^, ^27^, ^28^ From this set of research articles, comprising a period from 2016–2025, we manually identified names and targets from main figures of those immunotherapies and small molecules reaching Phase III, as well as searching related publications to determine the trial status (Table S1). This clinical stage of drug development was chosen because it represents the highest probability to be approved for commercialization after demonstrating safety, tolerability, and efficacy. The status of each drug was established mainly based on peer‐reviewed articles or data from ClinicalTrials.gov website. When peer‐reviewed data were limited, we established the status based on company communications, sponsor‐reported results discussed in press releases, investor materials, corporate websites, and others. Such non–peer‐reviewed references were interpreted with caution due to potential reporting bias and incomplete methodological disclosure. Of note, because this is a narrative review, it does not follow systematic or scoping review standards like Preferred Reporting Items for Systematic reviews and Meta‐Analyses (PRISMA). A summary of Phase III candidates (47 agents) reported in Cummings’ pipeline reports is presented in Figure 1.^19^, ^20^, ^21^, ^22^, ^23^, ^24^, ^25^, ^26^, ^27^, ^28^

The graph integrates Phase III timelines (gradient), mechanism‐of‐action classes (colored circles), and status (stars). Overall, 23.4% are disease modifying (DM) immunotherapies (DMI, *n* = 11) and 76.6% DM small molecules (DMSM, *n* = 36) including organic molecules and herbal extracts. From the DM agents (Table S2), 36.2% were mentioned in the first pipeline of 2016, 23.4% introduced during 2017–2020, and 40.4% were added between 2021 and Jan 1, 2025. However, only 38.3% (18 agents; 6 DMI and 12 DMSM) remain active in Phase III, whereas 61.7% (29 agents) were unsuccessful, discontinued, or lacked updates as of February 28, 2026 (Table S1, Figure 1).

As shown in Table S3, most drugs entering Phase III in 2016–2025 were anti‐Aβ (44.7%): 11 DMI and 11 DMSM. However, a considerable number of DMSM have as a target synaptic plasticity (19.1%), neuroprotection (17.0%), whereas other potential targets of candidates reaching Phase III were neuroinflammation (14.9%), metabolism and energetic balance (10.6%), and oxidative stress (8.5%). Most drugs entering Phase III in 2016–2017 were anti‐Aβ (84.2%): 6 DMI and 10 DMSM. Others included one anti‐tau, one anti‐inflammatory, and one metabolic modulator; two agents (ALZT‐OP1a/b, azeliragon) targeted both Aβ and neuroinflammation. During 2018–2020, mechanisms of candidates reaching Phase III were diverse. Only one anti‐Aβ immunotherapy (lecanemab), whereas the other 88.9% were small molecules with targets like synaptic plasticity (n = 5), neuroprotection (n = 5), metabolism and energetic balance (n = 2), neuroinflammation (n = 1), and vasculature (n = 1). Four drugs were multifunctional: icosapent ethyl (synaptic plasticity, neuroprotection, oxidative stress), blarcamesine (synaptic plasticity, neuroprotection), atuzaginstat (neuroinflammation, synaptic plasticity, neuroprotection), AGB101 (synaptic plasticity, neuroprotection), and troriluzole (synaptic plasticity, neuroprotection). Across 2016–2020, only six AD therapies were documented as active on Phase III trial (gantenerumab, lecanemab, masitinib, blarcamesine, metformin, tricaprilin); the others were discontinued due to adverse events, lack of efficacy, lack of updates or company decisions (see references in Table S1).

In the last 5 years, there were more DMSMs (78.9%, 15 agents) than DMIs (21.0%, 4 agents) entering Phase III trials. Anti‐Aβ agents reaching this stage by 2021‐2025 comprised two anti‐Aβ monoclonal antibodies (donanemab and remternetug), a DMSM (ALZ‐801) and a dual Aβ/tau regimen (lecanemab + E2814). Five agents were explicitly multi‐target: semaglutide (metabolism and energetic balance, neuroinflammation), simufilam (synaptic plasticity, neuroprotection, oxidative stress), fosgonimeton (synaptic plasticity, neuroprotection), and GV1001 (synaptic plasticity, neuroprotection), as well as lecanemab + E2814 (amyloid and tau pathologies). Others targeted single pathways: neuroinflammation (n = 2), oxidative stress (n = 2), metabolism/energetics (n = 1), neurogenesis (n = 1), others (n = 3), and one anti‐tau mAb (E2814). By 2025, only 18 of total candidates (38.3%) were found to be active, where 4 of them being multi‐target (8.5%).

Overall, the field has shifted from an Aβ‐only focus^29^ toward greater mechanistic diversity in the last 5 years. Although anti‐Aβ agents remain prominent in Phase III, indirect modulation of Aβ and tau via other pathways also appears capable of delaying cognitive decline. With the 2025 deadline for “effective therapy” now passed,^16^, ^17^ a key question remains: What are the lessons from 9 years of close pipeline tracking? Evidence reveals multiple vulnerabilities in AD; therefore, future strategies must be precise and multiplexed.

## 3 MULTI‐TARGET DRUGS FOR ALZHEIMER'S DISEASE

AD physiopathology becomes even more complex due to aging‐related changes plus additional pathological mechanisms, thereby complicating treatment.^30^, ^31^ Aging hallmarks linked to AD progression include genomic instability, macromolecular damage, epigenetic alterations, deregulated nutrient sensing, mitochondrial dysfunction, cellular senescence, impaired macroautophagy, stem cell exhaustion, altered intercellular communication, and dysbiosis. On the other hand, genetics also shapes progression and treatment response, as seen with certain Phase III candidates.^32^, ^33^, ^34^, ^35^ In addition, infections such as from *Porphyromonas gingivalis* may increase AD risk.^36^ Considering such events, effective therapies should therefore address multiple alterations, including Aβ and tau aggregation, inflammation, mitochondrial dysfunction, and synaptic/axonal defects.^31^

### 3.1 M1 muscarinic receptor agonists

Muscarinic receptors are G‐protein–coupled receptors activated by acetylcholine (ACh). They comprise five subtypes (M1–M5) and support vegetative, sensory, cognitive, and motor functions in the central nervous system.^37^ The M1 receptor is highly expressed in the cortex and hippocampus, representing ≈37% of muscarinic subtypes in rodents.^38^ In humans, M1 is the most abundant subtype in the cortex (frontal, temporal, parietal, and occipital areas; 35%–60%) and constitutes ≈60% of muscarinic receptors in the hippocampus, localized mainly in the pyramidal somata and apical and basal dendrites in the stratum radiatum and stratum oriens.^38^

Evidence for M1 agonists as potential AD treatments emerged in the early 2000s. At that time, acetylcholinesterase (AChE) inhibitors, such as donepezil, had shown that restoring cholinergic signaling improved cognition in AD,^39^ but these agents were symptomatic and did not modify underlying neurodegeneration. M1 stimulation was implicated in hippocampal–cortical cognitive processes.^40^ Unlike AChE inhibitors, M1 agonists were also reported to display neuroprotective effects and reductions of both brain and CSF Aβ levels of patients with AD.^41^ In the early 1980s, following the development of the neurotoxin AF64A,^42^ Fisher et al. investigated brain hypercholinergic signaling in neuropsychiatric disease and AD.^43^ Cognitive impairments in AF64A‐induced AD‐like rodents were reversed by low doses of the M1 agonist AF102B administered below side‐effect thresholds.^44^ AF102B and related “rigid” ACh analogues (AF150, AF151) showed high M1 selectivity, activating G proteins and increasing Ca^2^^+^ release, phosphatidylinositol hydrolysis, and arachidonic acid release without raising cAMP.^45^, ^46^ This signaling profile suggested mechanistic relevance, and AF102B was reported to lower total Aβ in the CSF of 19 patients with AD.^47^ Acute AF102B infusion also promoted non‐amyloidogenic APP processing over the amyloidogenic pathway, reducing CSF Aβ42.^48^ However, despite these findings, clinical development in AD was not pursued. AF150(S), which reversed AF64A‐induced deficits in rats^49^ and decreased tau hyperphosphorylation,^50^ did not progress to clinical trials.

Relative to AF102B and AF150(S), AF267B more effectively reduced Aβ peptides in rabbit CSF, enhanced non‐amyloidogenic α‐APP secretion in the hippocampus and cortex, promoted neurotrophin expression and dendritic growth, and protected PC12 cells (stably transfected with M1 muscarinic acetylcholine receptor) from Aβ‐induced apoptosis.^51^ Nevertheless, clinical development of these first‐generation M1 agonists for AD was suspended due to side effects (including gastrointestinal issues), low bioavailability, dosing constraints, and insufficient M1 selectivity (off‐target M2/M3 receptors activation).^52^ These limitations highlight the translational challenges associated with achieving central M1 selectivity while maintaining tolerability.

In 2003, introduction of the triple‐transgenic 3xTg‐AD mouse line (APP(Swe), presenilin PS1(M146V), tau(P301L)) ^53^ and subsequent studies using this mouse strain, clarified M1's role in AD pathophysiology.^54^ Four weeks of AF267B in 6‐months‐old female 3xTg‐AD mice improved learning and spatial memory, lowered Aβ_40/42_ in the cortex and hippocampus, and shifted APP processing toward non‐amyloidogenic pathways. Treatment also reduced ‐secretase expression, increased A disintegrin and metalloproteinase 17 (ADAM17) and phosphorylated epxtracellular signal‐regulated kinase 1 and 2 (ERK1/2), enhanced protein kinase C (PKC) activity, and inhibited tau hyperphosphorylation.^54^ Although these findings strengthened the mechanistic rationale for M1 stimulation, they remain confined to preclinical models until 2009, where Phase I results for AF267B were presented, indicating safety and good tolerability in healthy young and elderly volunteers.^52^ However, according to non–peer‐reviewed communications, these data have not appeared in a refereed journal as of July 2024—or February 2026—when this section was written.^55^ Although M1‐receptor stimulation remains biologically plausible in AD, its disease‐modifying potential in humans remains unclear and requires further rigorously controlled investigation.

### 3.2 Sigma‐1 receptor agonists

The sigma‐1 receptor (σ1R) is a multifunctional ≈24‐kDa chaperone widely distributed across tissues, including the brain,^56^ where it is highly expressed in microglia, mature and immature oligodendrocytes, and excitatory and inhibitory neurons.^57^, ^58^ σ1R localizes to mitochondria‐associated endoplasmic reticulum (ER) membranes (MAMs), plasma membrane, ER, nuclear membrane, mitochondrial membrane, and other compartments.^56^ The receptor was proposed by Martin et al. ^59^ and later characterized by Su^60^ via pharmacological binding studies with the synthetic opioid SKF‐10047 in guinea pig brain. Subsequent electrophysiological research showed that σ1R agonists potentiate hippocampal NMDA receptor responses,^61^ suggesting a crucial role in cognition.

Further studies demonstrated that low doses of σ1R ligands—including the (+)‐SKF‐10047 enantiomer but not (−)‐SKF‐10047—prevented MK‐801–induced memory impairment, supporting the hypothesis that σ1R agonists may modulate cognitive processes.^62^ The anti‐amnesic effects of σ1R ligands typically show a bell‐shaped dose‐response curve and involves modulation of both NMDA and nicotinic cholinergic systems.^63^, ^64^ Of note, amnesia induced by intracerebroventricular aggregated Aβ_25‐35_ in mice^65^ was attenuated by σ1R agonists and blocked by σ1R antagonists.^66^ On this basis, σ1R was proposed as a therapeutic target for neurodegenerative diseases such as AD.^67^ Characterization of σ1R signaling has also clarified ancillary actions of approved drugs: donepezil, an AChE inhibitor, binds σ1R with high affinity and behaves functionally as a σ1R agonist.^68^, ^69^ This interaction has been proposed as a possible contributor to additional benefits reported for donepezil, including reduced hippocampal lipid peroxidation after i.c.v. Aβ_25‐35_ ^69^ and, more recently, reduced Aβ load and microglial activation in the hippocampus of 3xTg‐AD mice.^70^ However, the extent to which σ1R engagement contributes to clinical outcomes in humans remains uncertain.

### 3.3 Dual M1/Sigma‐1 receptor agonists

Other molecules like antiepileptic compound tetrahydro‐N,N‐dimethyl‐5,5‐diphenyl‐3‐furanemethanamine (AE14), later named ANAVEX 1‐41,^71^ were found to be beneficial in AD via M1 muscarinic agonism.^72^ Subsequent studies revealed σ1R agonist activity linked to anti‐amnesic effects in mice subjected to i.c.v. administration of a phosphorothioate‐modified oligodeoxynucleotide (antisense therapy).^71^ The dual profile of AE14 as a nonselective muscarinic receptor (M1–M4) ligand and σ1R activator, enabled the prevention of Aβ_25–35_–induced memory impairment at 10–100 µg/kg, with mechanisms involving the attenuation of neuroinflammation, oxidative stress, and pyramidal‐cell loss.^73^

Another developed furan derivative referred as ANAVEX 2‐73 (tetrahydro‐N,N‐dimethyl‐2,2‐diphenyl‐3‐furanmethanamine hydrochloride; blarcamesine), also showed dual M1/σ1R agonism.^74^ Consistently, blarcamesine prevented cognitive deficits induced by scopolamine, dizocilpine, and Aβ_25‐35_ at very low doses (e.g., 300 µg/kg), potentially by improving redox responses to oxidative stress. Moreover, its metabolite, ANAVEX 19‐144, retained biological activity and prolonged effects.^74^ In the Phase IIb/III trial (NCT04314934, initiated in 2019 for patients with early AD), blarcamesine showed a favorable safety profile without neuroimaging adverse events and a significant slowing of cognitive decline and neurodegeneration.^34^

AF710B, also known as ANAVEX 3‐71, is another type of dual M1/σ1R agonist. At low concentrations, AF710B was neuroprotective—reducing markers of neurodegeneration, oxidative damage, Aβ accumulation, and tau hyperphosphorylation—and prevented cognitive dysfunction in pharmacologically induced rat models.^75^, ^76^ Moreover, long‐term treatment (4.5 months; 10 µg/kg) in 13‐month‐old McGill‐R‐Thy1‐APP rats, followed by 5 weeks off drug, alleviated cognitive deficits.^76^ Sustained cognitive benefits were accompanied by fewer plaques in the subiculum and cortex, lower CSF Aβ_42_, reduced Iba‐1 expression and Iba‐1^+^ cells in CA1 pyramidal layers, and increased cortical synaptophysin.^76^ Early intervention similarly prevented decline and reduced amyloid pathology, inflammation, and neurotrophic alterations in memory‐related regions.^77^ A Phase I study was approved in Australia in 2020,^78^ with subsequent publications reporting favorable pharmacokinetics and cardiovascular safety.^79^, ^80^

### 3.4 Dual anti‐Amyloid β/Sigma‐1 receptor agonist

As shown previously, multi‐target drugs such as dual M1/σ1R agonists may serve as complementary or alternative treatments to anti‐Aβ therapies.^34^ Yet, what would be the impact of a molecule combining σ1R agonist activity and anti‐Aβ properties on AD pathophysiology and progression? Development as well as clinical evidence for this class of multifunctional compounds is scarce and remains at preclinical stages.

As background, initial anti‐Aβ strategies involved small “chaperonin‐like” aggregation modulators inspired by natural chaperones such as heat‐shock proteins.^81^, ^82^ These low‐molecular‐weight compounds stabilize protein conformations under stress and inhibit misfolding as part of proteostasis mechanisms.^83^ Studies identified their potential to prevent protein aggregation in the brain,^84^, ^85^ and specific structural sites of amyloid peptides were proposed as targets to block oligomerization and fibrillization.^86^ Certain physicochemical features, such as Aβ hydrophobicity, contribute to aggregation and the toxicity of resulting oligomers.^87^ The hydrophobic domains of amyloid aggregates also facilitate binding of small molecules—such as nonsteroidal anti‐inflammatory drugs (NSAIDs) including diclofenac,^88^ naproxen,^89^ and ibuprofen.^90^ Building on this principle, the Laboratory of Neurochemistry at the Cuban Center for Neurosciences (CNEURO) developed a family of in silico and in vitro monosubstituted naphthalene‐derived compounds (Amylovis), radiolabeled for early AD detection^91^ and with therapeutic potential in conformational diseases and diabetes.^92^, ^93^, ^94^

These Amylovis compounds were designed to interact with a specific Aβ_42_ sequence (SGYEVHQKLVFF) and prevent conformational transitions required for aggregation. Their structure contains three core elements: (1) a naphthyl group enabling π–π stacking with aromatic residues; (2) an amidoalkyl chain providing hydrophilicity and hydrogen bonding sites; and (3) a variable terminal functional group that distinguishes each derivative.^95^ In silico analyses identified three candidates capable of disrupting fibril elongation and reducing fibrillar Aβ accumulation.^95^ Among them, CNEURO‐201 [methyl (2‐{[4‐(1‐naphthylamino)‐4‐oxobutanoyl]amino}ethyl)dithiocarbamate] formed stable complexes with Aβ42 monomers and fibrils while destabilizing aggregated structures—suggesting dual anti‐aggregation and disaggregation properties. Toxicity tests confirmed high safety margins.^95^ The compound inhibited Aβ42 aggregation in human microglia cultures, bound amyloid plaques ex vivo in transgenic mice, and reduced Aβ in the subiculum after 8 weeks of oral administration (1.0 mg/kg) in 12‐month‐old female 3xTg‐AD mice.^95^ Although initial findings hinted at cognitive benefits, subsequent studies confirmed that CNEURO‐201 slowed cognitive decline in AD rodent models.^96^, ^97^

On the other hand, the interaction between CNEURO‐201 and σ1R has been investigated through in silico, in vitro, and in vivo approaches.^96^ Molecular docking revealed π–π interactions between the naphthalene group and Tyr103 and van der Waals forces between the C = S bond region and His154—stabilizing the ligand–receptor complex. Functionally, CNEURO‐201 acted as a σ1R agonist, promoting dissociation of binding immunoglobulin protein (BiP) from σ1R with an half maximal inhibitory concentration (IC_50_) of 362 nM—greater potency than the reference ligand PRE‐084 (IC_50_ = 426 nM). This effect was blocked by the σ1R antagonist NE‐100.^96^ In vivo, CNEURO‐201 normalized hyperlocomotor activity in wolframin‐deficient (Wfs1ab knockout) zebrafish—an experimental model of Wolfram syndrome characterized by ER—mitochondrial dysfunction and calcium imbalance^98^—and reversed dizocilpine‐induced deficits in spontaneous alternation and passive‐avoidance tasks at 0.03–1 mg/kg (i.p.) and 3 µM. These effects were also abolished by NE‐100.^96^

In cell‐free systems, CNEURO‐201 completely prevented and reversed Aβ aggregation in a concentration‐dependent manner.^96^ Other σ1R ligands, such as PRE‐084 and NE‐100, only partially prevented aggregation and achieved ≈25% disaggregation without dose dependence. These findings demonstrate a specific anti‐Aβ action of CNEURO‐201,^95^, ^97^ attributed to its optimized interaction with β‐sheets of Aβ. Thus, across chronic transgenic and acute pharmacological models, CNEURO‐201 exerts dual neuroprotection—via σ1R agonism and direct modulation of Aβ aggregation/disaggregation. Recent studies confirmed that low‐dose CNEURO‐201 (0.1–1 mg/kg) clears existing Aβ plaques in vivo in 3xTg mice and reduces glial activation.^97^ These effects may arise from σ1R activation in glial cells—known to express the receptor abundantly^57^, ^58^—modulating intracellular Ca^2^^+^ signaling, migration, and Aβ clearance.^99^ In addition, plaque disaggregation may attenuate neuroinflammation by decreasing glial reactivity. At similar doses, CNEURO‐201 also enhanced cholinergic transmission, acting as a mild AChE inhibitor that limited ACh degradation in hippocampus and cortex (in silico and in vivo). ^97^ However, aggregation–disaggregation mechanisms raise important mechanistic considerations, including the potential transient increase of soluble oligomeric intermediates, which warrants further investigation.

Overall, the preclinical evidence of CNEURO‐201 suggests multi‐target neuroprotection that may slow cognitive decline in AD. Nevertheless, all available data derive from experimental models, and no clinical trials have yet evaluated safety, pharmacokinetics, or efficacy in humans. Although no clinical trials are yet active, CNEURO‐201 is an example of convergence of efforts of international scientific research cooperation between Latin American and European academic institutions for future translational development focused on multi‐target molecules for AD and related dementias.

### 3.5 Other potential multi‐target drugs

Multi‐target drug development for AD has progressed to repurpose existing molecules and design compounds that simultaneously modulate multiple interconnected pathological processes, aiming to overcome the limited efficacy of single‐target treatments by integrating amyloid, tau, inflammatory, metabolic, and synaptic mechanisms.^100^, ^101^ In this context, AD‐associated biomarkers have been instrumental in categorizing candidate therapeutics according to their primary targets using the National Institute on Aging/Alzheimer's Association (NIA/AA) Common Alzheimer's Disease Research Ontology (CADRO) system.^102^ This updated framework revealed that, as of 2025, only about 4% of drugs in clinical trials exhibit multitarget mechanisms,^28^ underscoring both the challenges these compounds pose for drug development and their potential therapeutic value (Table 1).

**TABLE 1:** Compounds in advanced clinical stages or available exemplifying AD drug development shift toward multi‐target strategies.

Drug	Primary Target / Mechanism	Neuroprotective / Cognitive Effects	Additional Actions	References
Buntanetap (Posiphen / ANVS401)	↓ Translation of APP and α‐synuclein; ↓ neuroinflammation	↑ Cognitive performance, ↓ Aβ burden, ↑ neuronal integrity	Multifunctional: ↓ pro‐inflammatory cytokines, potential efficacy in Parkinson's disease	^103^, ^104^, ^105^
Masitinib	Tyrosine kinase inhibitor targeting microglia, mast cells, Aβ, and tau	↓ Cognitive decline, ↑ synaptic plasticity and dendritic spine density	↓ Microglial and mast cell activation; neuroimmune modulation	^106^, ^107^
ACD856	Positive allosteric modulator of TrkA/B/C (BDNF, NGF pathways)	↑ Synaptic connectivity, ↑ neuronal viability, ↑ mitochondrial performance	↓ IL‐6 and PGE_2_, ↑ neurogenesis, ↓ inflammation	^108^, ^109^, ^110^, ^111^
SDI‐118	Modulator of SV2A regulating vesicular neurotransmitter release	↑ Synaptic efficiency, ↑ vesicular recycling, ↑ calcium‐dependent priming	↑ Mitochondrial fusion/fission balance; improved neurotransmission	^112^, ^113^, ^114^, ^115^
NE3107	Anti‐inflammatory and insulin‐sensitizing (ERK/NF‐κB pathway)	↑ Cognitive and metabolic function, ↓ neuroinflammation	↓ Oxidative stress; restores neuroimmune and metabolic balance	^116^, ^117^, ^118^
Donepezil	Acetylcholinesterase inhibitor and neuroprotective properties attributed to its interaction with σ1R	↑ Cognitive function, ↑ Cholinergic signaling	↑ Long‐term potentiation; ↑ oligodendrocyte differentiation and remyelination; ↑ neuroprotection against ischemia‐induced hippocampal damage; cardio‐ and hepatoprotective effects against ER stress induced by high‐fat diets	^77^, ^119^, ^120^, ^121^, ^122^, ^123^
GV‐971	Modulation of gut microbiota	Anti‐inflammatory, ↓ Aβ burden	Sex‐dependent effects	^124^, ^125^

Abbreviations: ↓ indicates decrease or reduction; APP, amyloid precursor protein; Aβ, amyloid β; BDNF, brain‐derived neurotrophic factor; ERK, extracellular signal‐regulated kinase; IL‐6, interleukin‐6; NF‐κB, nuclear factor kappa‐B; NGF, nerve growth factor; PGE_2_, prostaglandin E2; SV2A, synaptic vesicle glycoprotein 2A; σ1R, sigma‐1 receptor; ER, endoplasmic reticulum;Trk, tropomyosin receptor kinase; ↑ indicates increase or enhancement.

### 3.6 Beyond Alzheimer's disease

An important feature of σ1R agonists is its nodal signaling can extend its application to other brain diseases. For instance, in a Phase 2 trial, AF710B reported positive safety and tolerability in adults with schizophrenia.^126^ Preclinical findings of blarcamesine have shown certain potential against Methyl‐CpG Binding Protein 2 (MECP2)‐related conditions in rodent models of Rett syndrome^127^ and Fragile X syndrome.^128^ Company communications have also referenced applications of blarcamesine for epilepsy,^129^ infantile spasms,^130^ Angelman syndrome,^131^ and Parkinson's disease dementia.^132^ To date, blarcamesine has Orphan Drug Designation (ODD) for Rett, Fragile X, and Angelman syndromes,^133^ and a 2024 U.S. patent covers blarcamesine and analogues for multi‐indication therapy.^134^ Similarly, AF710B received ODD for frontotemporal dementia in 2016.^135^ According to the company's website,^136^ current trials for blarcamesine include Alzheimer's disease (Phase III), Parkinson's disease dementia (Phase II), Rett syndrome (Phase II/III), Fragile X syndrome (Phase II/III), infantile spasms (Phase I), Angelman syndrome (Phase I), and an undisclosed rare disease (Phase I). Planned Phase II/III studies for AF710B include frontotemporal dementia and Alzheimer's disease. Of note, because some of these updates rely on corporate disclosures rather than peer‐reviewed publications, independent confirmation remains necessary.

Furthermore, aggregation‐prone peptides such as amylin (IAPP) also play roles in metabolic and neurodegenerative conditions. During early type 2 diabetes mellitus (T2DM), amylin misfolds into cytotoxic oligomers and fibrils that trigger apoptosis.^137^ Amylin deposits have been detected in the cerebral microvasculature of AD patients and animal models, linking T2DM and AD pathology.^138^ CNEURO‐201 binds the amylin 11‐28 region via hydrogen bonds, hydrophobic, and van der Waals interactions, inhibiting fibril formation and preventing apoptosis in cerebellar granule neurons.^94^ In streptozotocin‐induced diabetic models, CNEURO‐201 reduced hippocampal neuronal loss and improved spatial memory,^139^ effects likely associated with modulation of insulin‐signaling kinases involved in neuronal plasticity. These cross‐disease observations further expand the mechanistic rationale but remain preclinical and exploratory. In addition, stimulation of σ1R can prevent diabetes‐associated cognitive dysfunction.^140^, ^141^

Collectively, these findings indicate that stimulating σ1R signaling is biologically plausible as a therapeutic strategy for AD and potentially other dementias, neuropsychiatric illnesses, or neurological disorders. Moreover, positron emission tomography (PET)/magnetic resonance imaging (MRI) data from early AD suggests increased σ1R expression as an early biomarker.^142^ Responses to σ1R agonists in AD may be influenced by genetics, for example, the SIGMAR1 rs1800866 variant,^34^ which underscores the need to further investigate σ1R biology in brain disease. Moreover, further large, independently validated trials could help in the translation of these mechanistic insights into consistent and clinically meaningful outcomes.

### 3.7 Limitations of multitarget drugs

A rational strategy for addressing the complex pathology of AD involves the development of multi‐target drugs. However, among the 25.5% of multitarget candidates that entered Phase III trials between 2016 and 2025, only four remain active (Figure 1, Table S1). This low retention rate suggests that multifunctional therapeutic strategies in AD face substantial limitations and developmental challenges. These difficulties become evident when attempting to translate the biological complexity of AD (including cholinergic dysfunction, amyloid aggregation, tau hyperphosphorylation, oxidative stress, metal dyshomeostasis, and monoaminergic imbalance), into a single pharmacological entity. Such an approach inevitably introduces both conceptual and operational tensions.

Although achieving balanced polypharmacology is a central objective, it is important to recognize that the relative contribution of these pathophysiological processes varies across preclinical, prodromal, and advanced stages of AD. Designing a single compound capable of simultaneously modulating all altered pathways may therefore oversimplify the temporal and biological heterogeneity observed in patients. It is important to note that the assumption that concurrent modulation of multiple targets necessarily produces synergistic clinical benefits remains insufficiently demonstrated in human studies. Moreover, broader interaction profiles in DMSMs may increase the likelihood of off target effects, whereas simultaneous modulation of multiple pathways could generate additive adverse responses that are difficult to attribute mechanistically.

Additional complexity emerges from regulatory and translational perspectives. Drug approval pathways are traditionally structured around clearly defined primary mechanisms of action, target‐specific endpoints, and the isolated attribution of adverse effects. In contrast, multi‐target agents operate through coordinated modulation of multiple biological systems, which can complicate mechanistic interpretation, endpoint selection, and safety assessment. As noted previously in discussions of multi‐target drug development,^143^, ^144^ evaluation of such compounds may require composite biomarkers, stage‐stratified patient selection, and outcome measures capable of capturing network‐level effects. Although these methodological adaptations may provide a more accurate representation of drug activity, they also introduce additional layers of complexity that may hinder clinical translation.

Nonetheless, despite these limitations, the evidence discussed in this work suggests that certain compounds with agonistic activity at M1 or sigma‐1 receptors may exert multifunctional effects through the focused engagement of key regulatory nodes. Rather than attempting broad and indiscriminate pathway coverage, selective modulation of nodal signaling systems may influence multiple downstream pathological processes in a network‐dependent manner. Within this integrative neurobiological framework, strategic single‐point interventions directed at biologically central targets could potentially produce pleiotropic effects without requiring simultaneous direct modulation of all pathological mechanisms. Consequently, establishing clear criteria for defining and evaluating compounds based on their nodal signaling properties may become increasingly important. Nevertheless, whether such nodal targeting can consistently translate into clinically meaningful and durable therapeutic benefits remains to be demonstrated through rigorously designed and independently validated clinical studies.

## 4 NON‐PHARMACOLOGICAL MULTI‐TARGET STRATEGIES

Non‐pharmacological interventions have emerged as essential, complementary components of therapeutic management.^145^, ^146^ These approaches, based primarily on healthy lifestyle modifications, can modulate multiple pathological processes while remaining accessible, low‐risk, and cost‐effective compared to conventional pharmacotherapies. Interventions such as physical exercise, dietary modification, sleep optimization, and cognitive stimulation have demonstrated multi‐target effects involving neuroinflammation, oxidative stress, synaptic plasticity, and cerebral energy metabolism.

### 4.1 Physical exercise

Extensive clinical and preclinical evidence supports physical exercise as a beneficial intervention in AD. Exercise reduces cerebral Aβ burden and hyperphosphorylated tau, enhances cerebral blood flow, promotes neurogenesis, and stimulates the release of neurotrophic factors and neurotransmitters. It further improves brain plasticity and attenuates oxidative stress, inflammation, and atrophy.^147^, ^148^ These effects correlate with enhanced memory, attention, executive function, processing speed, and overall cognitive and functional performance, contributing to improved quality of life across disease stages.^149^ The beneficial effects of exercise are largely mediated by myokines, including irisin and cathepsin B. Irisin, derived from the precursor fibronectin type III domain‐containing protein 5 in skeletal muscle, crosses the blood–brain barrier and induces hippocampal expression of brain‐derived neurotrophic factor (BDNF), promoting neurogenesis, synaptogenesis, and cognitive improvement. Cathepsin B, also induced by exercise, contributes to amyloid degradation and neurogenic signaling; its elevation is associated with improved spatial memory and reduced neuropathological markers.^150^, ^151^ These findings highlight the role of skeletal muscle as an endocrine organ supporting brain function. Although standardized exercise prescriptions for patients with AD are lacking, experts recommend 150–300 min per week of moderate physical activity combined with multimodal interventions encompassing aerobic, resistance, flexibility, and coordination training.^103^, ^152^ Exercise represents a safe, multitarget therapeutic approach that may act synergistically with pharmacological treatments to enhance neuroprotection.

### 4.2 Nutrition, supplementation, and nutraceuticals

Dietary interventions and nutritional supplementation (Table 2) have gained relevance as integrative strategies capable of modulating several pathological mechanisms underlying AD,^104^ which should be personalized according to clinical and nutritional profiles.

**TABLE 2:** Nutritional strategies with multifunctional beneficial properties in AD.

Type of intervention	Main Components/Mechanism	Neuroprotective and Cognitive Effects	Underlying Pathways/Biomarkers	References
Mediterranean diet	Antioxidants and PUFA‐rich foods (olive oil, nuts, fish)	↓ Cognitive decline, ↑ memory, attention, executive function	↓ Inflammation and oxidative stress; ↑ BBB integrity	^153^
Ketogenic diet	↓ Carbohydrates → ↑ Ketone bodies as alternative energy source	↑ Mitochondrial efficiency, ↑ synaptic plasticity, ↓ neuroinflammation	↑ ATP generation, ↓ ROS, ↓ NF‐κB signaling	^154^
MIND diet	Combines Mediterranean and DASH patterns	↑ Hippocampal volume, ↑ BDNF, ↑ cognition	↑ SIRT1 and neuroprotective gene activation	^155^
Creatine	Enhances phosphocreatine–creatine kinase system	↑ ATP availability, ↓ oxidative damage, ↑ synaptic function	↑ Mitochondrial buffering, ↓ ROS	^156^, ^157^
Omega‐3 fatty acids (DHA, EPA)	Modulate cytokines (↓ TNF‐α, ↓ IL‐6)	↓ Cognitive decline, ↑ antioxidant capacity	↓ Inflammation, ↑ neuronal membrane fluidity	^158^
Vitamins and minerals	B1, B6, B9, B12, C, E, selenium, zinc	↓ Neurotoxicity, ↑ enzymatic and mitochondrial function	↓ Brain atrophy, ↑ cognition	^159^
Nutraceuticals	Curcumin, resveratrol, flavonoids	↓ Aβ and tau aggregation, ↑ autophagy, ↑ antioxidant capacity	↑ Nrf2, ↑ SIRT1, ↓ inflammation	^160^

Abbreviations: ↓ indicates decrease or reduction; Aβ, amyloid beta; BBB, blood^–^brain barrier; BDNF, brain‐derived neurotrophic factor; DHA, docosahexaenoic acid; EPA, eicosapentaenoic acid; IL‐6, interleukin‐6; NF‐κB, nuclear factor kappa‐B; Nrf2, nuclear factor erythroid 2‐related factor 2; PUFA, polyunsaturated fatty acids; ROS, reactive oxygen species; SIRT1, sirtuin 1; TNF‐α, tumor necrosis factor alpha; ↑ indicates increase or enhancement.

### 4.3 Non‐invasive brain stimulation

Non‐invasive brain stimulation techniques, including repetitive transcranial magnetic stimulation and transcranial direct current stimulation, have shown the potential to enhance cognitive performance—specifically verbal memory, attention, learning, processing speed, and working memory. Mechanistically, non‐invasive brain stimulation increases neurotransmitter release (e.g., dopamine), facilitates synaptic plasticity, and regulates the hypothalamic–pituitary–adrenal axis.^105^, ^106^ Other modalities—transcranial alternating current stimulation, transcranial random noise stimulation, and 40 Hz auditory/visual stimulation—also promote cognitive improvement and clearance of pathological proteins.^107^, ^108^, ^109^ Further research is required to standardize stimulation parameters and assess synergistic combinations with pharmacological and lifestyle interventions.

### 4.4 Acupuncture

Acupuncture has been investigated as an adjunctive therapy for AD. Clinical and experimental evidence indicates improvements in memory, attention, learning, and executive function (e.g., Mini‐Mental State Examination [MMSE] scores), alongside reductions in insomnia, anxiety, and depression.^110^ Mechanisms include inhibition of neuroinflammation (via regulation of interleukin 1β and tumoral necrosis factor α), attenuation of oxidative stress through modulation of antioxidant enzymes (superoxide dismutase, catalase), enhancement of mitochondrial function, and normalization of cholinergic transmission. Acupuncture also reduces amyloid and tau accumulation in the hippocampus and cortex,^111^, ^112^ supporting its characterization as a multi‐target therapeutic approach.

### 4.5 Photobiomodulation

Photobiomodulation (PBM) refers to the therapeutic application of non‐ionizing and nonthermal light with the aim of modulating biological processes through photochemical and photophysical changes. PBM has been proposed as an AD non‐pharmacological intervention intended to restore cerebral bioenergetics, modulate neuroinflammation, and promote clearance pathways of pathological proteins, conceptually integrating into a multitarget therapeutic framework to stimulate cortical networks involved in memory and executive functions.^113^, ^114^, ^115^ Protocols and devices in AD are usually configured for wavelengths in the red and near infrared range, typically between 630 and 1070 nm.^113^, ^115^

In preclinical models, irradiation at 1070 nm has been shown to improve cognitive performance and reduce Aβ burden, in association with microglial modifications and vascular components involved in clearance, suggesting that PBM may increase the resolution capacity of the cerebral microenvironment.^116^ Complementarily, PBM has been reported to induce intercellular changes mediated by exosomes derived from light modulated microglia, with impact on synaptic integrity and cognition in AD models.^117^ On the other hand, clinical studies in AD patients suggest that PBM may improve executive domains, working memory, and verbal components, with an acceptable safety profile and high adherence to protocols.^113^, ^118^ However, robust controlled clinical trials are required, together with correlation of these effects with disease‐related pathological biomarkers and standardization of safe and effective dose parameters according to disease stage.

### 4.6 Sleep, meditation, and music

Lifestyle‐centered interventions, such as sleep optimization, meditation, and music therapy, contribute to neurocognitive resilience. Adequate sleep facilitates Aβ clearance and circadian regulation, enhancing attention, memory consolidation, and emotional balance.^119^ Meditation and mindfulness improve working memory and attention while reducing anxiety and depression through decreased cortisol levels, reduced oxidative stress, and enhanced neuronal plasticity.^120^ Music therapy activates limbic, frontal, and temporal regions, increases neural connectivity, and promotes the release of dopamine, serotonin, and oxytocin, thereby improving memory, language, social interaction, and mood.^121^

### 4.7 Limitations of non‐pharmacological interventions

Despite their therapeutic potential, non‐pharmacological interventions remain underutilized or insufficiently integrated into standard clinical guidelines. Promoting their implementation and evaluating their synergistic effects in combination with pharmacological therapies may help address the multifactorial pathophysiology of AD. However, non‐pharmacological interventions present several limitations. First, an important limitation is the heterogeneity of intervention protocols and outcome measures, including differences in training intensity, modality, duration, and adherence monitoring, as well as the limited availability of standardized protocols specifically designed for individuals with AD and adapted according to disease stage or degree of motor impairment. In the case of dietary interventions, dietary patterns, food availability, and variability in formulations represent additional challenges for the broad or universal implementation of this strategy. The implementation of neuromodulatory techniques also faces significant methodological variability, particularly regarding stimulation parameters, treatment duration, and patient selection criteria. Interpretation of clinical outcomes in acupuncture therapy is often complicated by differences in stimulation protocols, acupuncture points, treatment frequencies, and practitioner‐dependent variability. In PBM, the absence of standardized dosimetry and treatment protocols complicates the identification of optimal therapeutic windows and limits the comparability of studies. Finally, isolating specific biological mechanisms or quantifying long‐term disease‐modifying effects of relaxation‐based activities, such as meditation or music therapy, remains difficult because their outcomes are highly sensitive to behavioral and environmental variables.

## 5 SYNERGISTIC OPTIMIZED PHARMACOLOGICAL AND HOLISTIC INTERVENTIONS FOR ALZHEIMER'S DISEASE

**FIGURE 2:**
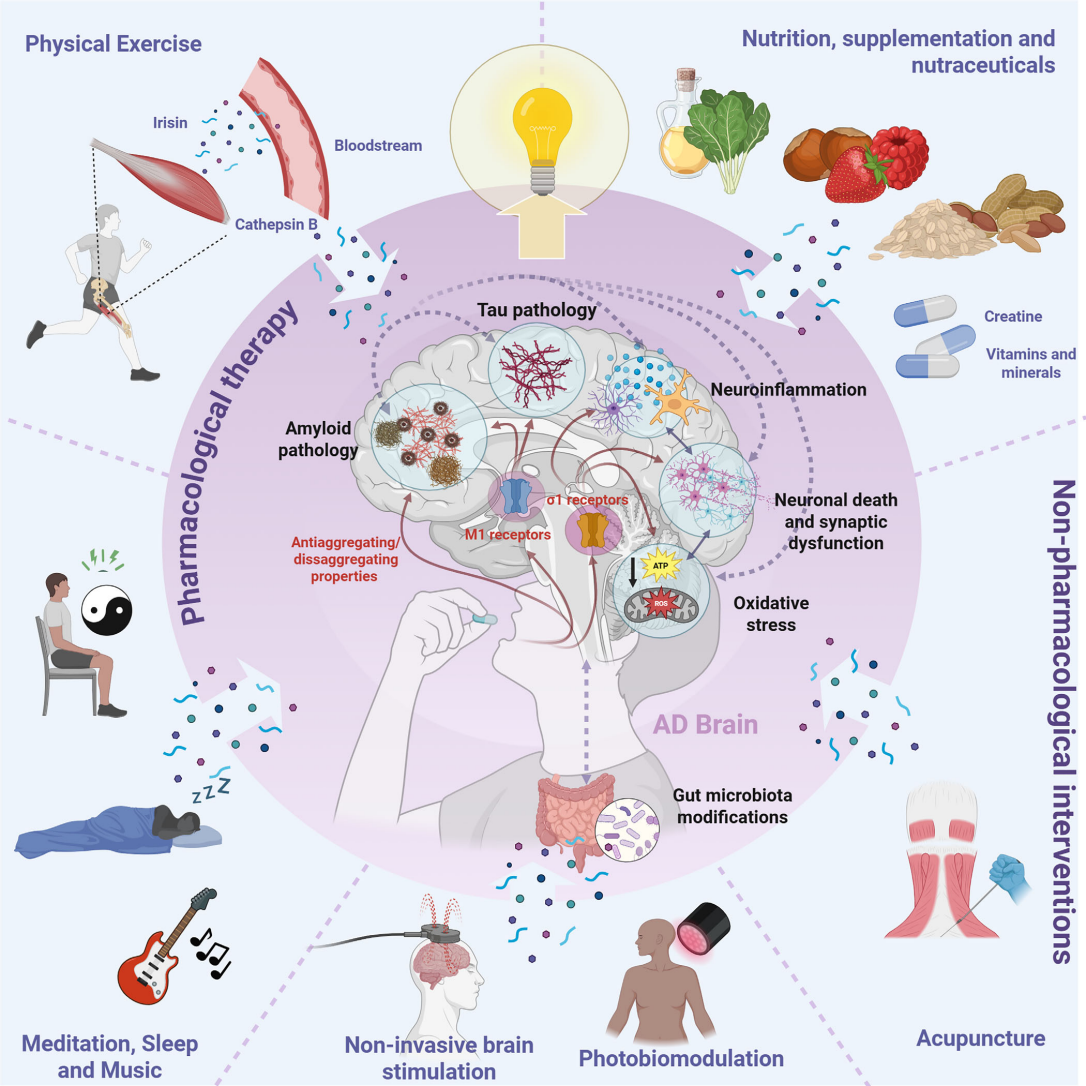
Synergistic Optimized Pharmacological and Holistic Interventions for Alzheimer's Disease (SOPHI‐AD) to effective slowing of cognitive decline. Integration of multitarget pharmacological and non‐pharmacological approaches against pathophysiological mechanisms underlying AD (Illustration created in BioRender).

Despite advances in pharmacological research, current treatments for AD provide only modest benefits and fail to prevent disease progression. This limitation has prompted the development of a more integrative therapeutic framework, termed Synergistic Optimized Pharmacological and Holistic Interventions for Alzheimer's Disease (SOPHI‐AD) (Figure 2). SOPHI‐AD integrates pharmacological and non‐pharmacological approaches that complement each other in targeting the multiple, interrelated pathophysiological mechanisms underlying AD. Multi‐target pharmacological agents act on key processes such as neuroinflammation, oxidative stress, mitochondrial dysfunction, and synaptic plasticity, thereby supporting cognitive preservation.^12^ In parallel, non‐pharmacological interventions—including physical exercise, dietary optimization, and non‐invasive brain stimulation—exert multifaceted effects capable of influencing the same molecular and cellular pathways, producing synergistic outcomes that enhance pharmacological efficacy.^145^

Human studies have demonstrated the potential of non‐pharmacological interventions to produce effects comparable to pharmacological treatments. For instance, regular physical activity yielded cognitive benefits similar to those of donepezil in AD patients with mild cognitive impairment.^122^ Moreover, a 20‐week multimodal lifestyle intervention produced significant cognitive improvements in AD patients in a controlled clinical trial.^123^ These findings suggest that programs including SOPHI‐AD may provide superior outcomes compared to pharmacological or non‐pharmacological therapies alone.

Mechanistic convergence further supports this framework. Certain multi‐target drugs and physical exercise increase BDNF expression,^124^, ^143^ enhancing synaptic plasticity and neuronal connectivity. Similarly, antioxidant‐rich diets and specific pharmacological agents mitigate oxidative damage, paralleling the antioxidative effects of exercise.^125^, ^161^ In addition, non‐invasive brain stimulation, when combined with cognitive training, enhances neurotransmitter release and strengthens cortical circuits involved in cognition.^106^ Thus, combining multiple non‐pharmacological therapies within the SOPHI‐AD model could potentiate pharmacological treatment effects through shared molecular pathways. Collectively, these overlapping mechanisms support the hypothesis that SOPHI‐AD may maximize therapeutic benefits, slow disease progression, and help preserve functional independence and quality of life. Understanding and optimizing such convergent mechanisms represents a central objective of multi‐target therapeutic design.

Despite robust scientific evidence supporting the SOPHI‐AD framework, several challenges hinder its clinical implementation. Standardized protocols defining optimal dosages, timing, and combinations of interventions across different AD stages remain lacking. Furthermore, despite demonstrated efficacy, non‐pharmacological therapies are still underrepresented in clinical practice guidelines as adjunctive strategies. Additional barriers include insufficient multidisciplinary collaboration and limited integration within public health systems. This is particularly important, since elucidation of mechanisms and evaluation of the impact on pathophysiology of SOPHI‐AD frameworks in AD patients can be a complex task, requiring experts from different fields including nutritionists, physiotherapists, clinical specialists, physicists, data scientists, geneticists, psychologists, and experts in complementary and alternative medicine. In the short term, overcoming these limitations will require targeted awareness efforts, the development of international consensus on multimodal intervention protocols, and the systematic incorporation of combined pharmacological and lifestyle‐based therapies into health care and prevention programs.

## 6 CONCLUSION

AD therapies continue to pose a complex challenge. Despite major advances in understanding its multifactorial biology, therapeutic progress remains modest, with ongoing reflection on the difficulty of translating mechanistic insights into durable clinical benefit. The diversification of targets observed in recent drug‐development pipelines indicates a gradual shift away from single‐pathway approaches toward more integrated therapeutic concepts. Multi‐target pharmacological strategies, particularly those acting on nodal signaling systems such as M1 and sigma‐1 receptors, provide a biologically plausible framework for influencing multiple downstream pathological processes. Nevertheless, substantial translational challenges remain. Moreover, complementary non‐pharmacological interventions such as physical exercise, nutritional strategies, neuromodulation, and lifestyle optimization may further contribute to synergistically modulate in a positive manner the complex network of mechanisms involved in AD. Within this context, the SOPHI‐AD framework is proposed as a conceptual platform for future investigation, emphasizing the potential value of coordinated pharmacological and holistic interventions. Ultimately, advancing AD therapy will likely require rigorously validated multimodal strategies capable of addressing the dynamic and heterogeneous nature of the disease.

## AUTHOR CONTRIBUTIONS

All authors contributed to the study conception and design. Data collection and revision were performed by H.M.O, C.R.T., J.A.G., and N.J.P.C. The first draft of the manuscript was written by H.M.O and revised by S.Y.D.M and C.R.T., and all authors commented on previous versions of the manuscript. The tables and figures were made by H.M.O, J.A.G., and N.J.P.C. All authors read and approved the final manuscript.

## CONFLICT OF INTEREST STATEMENT

The authors declare no competing interests. Author disclosures are available in the Supporting Information.

## Supporting information




**Supporting Information**: alz71589‐sup‐0001‐SupMat.docx

**Supporting Information**: alz71589‐sup‐0002‐ICMJE.pdf

## Data Availability

No datasets were generated or analyzed during the current study.
