# Supplementary material for: Redirecting Alzheimer's disease therapeutics: Multitarget drugs and complementary non‐pharmacological strategies
Source: Alzheimers Dement. 2026 Jun 23;22(6):e71589. doi: 10.1002/alz.71589 (PMC13290666; doi:10.1002/alz.71589)
Supplement: Supplementary file 1 — Supporting Information: alz71589‐sup‐0001‐SupMat.docx [file ALZ-22-e71589-s001.docx]

**Supplementary Table 1. Alzheimer’s disease drug candidates currently in Phase III clinical trials. Data compiled from the 2016–2025 Alzheimer’s Disease Drug Development Pipeline Reports by Cummings et al.**

| **Therapy classification** | **No.** | **Drug description** | **First introduction as Phase III trial molecule in the pipeline reports*** | **Related target(s)** | **Last mention as Phase III trial molecule in the pipeline reports*** | **Status** | **References** |
| --- | --- | --- | --- | --- | --- | --- | --- |
| DMI | 1 | Albumin+IVIG | 2016 | Amyloid pathology | 2019 | Inactive/Discontinued/Without updates | [1] |
|  | 2 | CAD106 | 2016 | Amyloid pathology | 2020 | Inactive/Discontinued/Without updates | [2] |
|  | 3 | Gantenerumab | 2016 | Amyloid pathology | 2025 | Active | [3] |
|  | 4 | Solanezumab | 2016 | Amyloid pathology | 2025 | Inactive/Discontinued/Without updates | [4-6] |
|  | 5 | Aducanumab | 2016 | Amyloid pathology | 2024 | Inactive/Discontinued/Without updates | [7, 8] |
|  | 6 | Crenezumab | 2017 | Amyloid pathology | 2020 | Inactive/Discontinued/Without updates | [9, 10] |
|  | 7 | Lecanemab | 2020 | Amyloid pathology | 2025 | Active | [11] |
|  | 8 | Donanemab | 2022 | Amyloid pathology | 2025 | Active | [12] |
|  | 9 | Remtemetug | 2023 | Amyloid pathology | 2025 | Active | [13] |
|  | 10 | E2814 (Etalanetug) | 2023 | Tau pathology | 2025 | Active | [14] |
|  | 11 | E2814+Lecanemab | 2025 | Amyloid pathology, tau pathology | 2025 | Active | [15] |
| DMSM | 1 | Verubecestat (MK-8931) | 2016 | Amyloid pathology | 2018 | Inactive/Discontinued/Without updates | [16] |
|  | 2 | Sodium oligomannate (GV-971) | 2016 | Amyloid pathology | 2022 | Inactive/Discontinued/Without updates | [17, 18] |
|  | 3 | Umibecestat (CNP520) | 2016 | Amyloid pathology | 2019 | Inactive/Discontinued/Without updates | [19] |
|  | 4 | ALZT-OP1a/b (Cromolyn Sodium + ibuprofen) | 2016 | Amyloid pathology, neuroinflammation | 2020 | Inactive/Discontinued/Without updates | [20] |
|  | 5 | Nilvadipine | 2016 | Amyloid pathology | 2017 | Inactive/Discontinued/Without updates | [21] |
|  | 6 | Lanabecestat (AZD3293/LY3314814) | 2016 | Amyloid pathology | 2018 | Inactive/Discontinued/Without updates | [22] |
|  | 7 | Azeliragon (TTP488) | 2016 | Amyloid pathology,neuroinflammation | 2021 | Inactive/Discontinued/Without updates | [23-25] |
|  | 8 | Atabecestat (JNJ-54861911) | 2016 | Amyloid pathology | 2018 | Inactive/Discontinued/Without updates | [26, 27] |
|  | 9 | Pioglitazone | 2016 | Amyloid pathology | 2017 | Inactive/Discontinued/Without updates | [28, 29] |
|  | 10 | Insulin | 2016 | Metabolism and energetic balance | 2018 | Inactive/Discontinued/Without updates | [30] |
|  | 11 | Masitinib | 2016 | Neuroinflammation | 2025 | Active | [31] |
|  | 12 | Elenbecestat (E2609) | 2017 | Amyloid pathology | 2019 | Inactive/Discontinued/Without updates | [32] |
|  | 13 | Icosapent ethyl (Vascepa, Eicosapentaenoic Acid) | 2018 | Synaptic plasticity, neuroprotection, oxidative stress | 2023 | Inactive/Discontinued/Without updates | [33] |
|  | 14 | ANAVEX 2-73 (Blarcamesine) | 2019 | Synaptic plasticity, neuroprotection | 2025 | Active | [34] |
|  | 15 | Atuzaginstat (COR388) | 2019 | Neuroinflammation, synaptic plasticity, neuroprotection | 2022 | Inactive/Discontinued/Without updates | [35, 36] |
|  | 16 | Losartan+Amlodipine+Atorvastatin | 2019 | Vasculature | 2022 | Inactive/Discontinued/Without updates | [37, 38] |
|  | 17 | Levetiracetam (AGB101) | 2019 | Synaptic plasticity, neuroprotection | 2024 | Inactive/Discontinued/Without updates | [39-41] |
|  | 18 | Troriluzole (BHV4157) | 2019 | Synaptic plasticity, neuroprotection | 2021 | Inactive/Discontinued/Without updates | [42] |
|  | 19 | Metformin | 2020 | Metabolism and energetic balance | 2025 | Active | [43] |
|  | 20 | Tricaprilin | 2020 | Metabolism and energetic balance | 2025 | Active | [44, 45] |
|  | 21 | Bezisterim (NE3107) | 2021 | Neuroinflammation | 2023 | Inactive/Discontinued/Without updates | [46, 47] |
|  | 22 | Omega 3 | 2021 | Oxidative stress | 2023 | Inactive/Discontinued/Without updates | [48] |
|  | 23 | Hydralazine Hydrochloride | 2022 | Oxidative stress | 2023 | Inactive/Discontinued/Without updates | [49, 50] |
|  | 24 | Nilotinib | 2022 | Other pathways | 2025 | Active | [51] |
|  | 25 | Semaglutide | 2022 | Metabolism and energetic balance, neuroinflammation | 2025 | Active | [52, 53] |
|  | 26 | Simufilam | 2022 | Synaptic plasticity, neuroprotection, oxidative stress | 2025 | Inactive/Discontinued/Without updates | [54] |
|  | 27 | Valiltamiprosate (ALZ-801) | 2022 | Amyloid pathology | 2025 | Active | [55, 56] |
|  | 28 | Fosgonimeton | 2023 | Synaptic plasticity, neuroprotection | 2024 | Inactive/Discontinued/Without updates | [57] |
|  | 29 | Piromelatine | 2023 | Other pathways | 2023 | Inactive/Discontinued/Without updates | [58] |
|  | 30 | Tertomotide (GV1001) | 2023 | Synaptic plasticity, neuroprotection, neurogenesis | 2025 | Active | [59] |
|  | 31 | Buntanetap | 2024 | Other pathways | 2025 | Active | [60] |
|  | 32 | PM012 | 2024 | Neurogenesis | 2025 | Inactive/Discontinued/Without updates | No data was found |
|  | 33 | GnRH Therapy (Gonadorelin acetate) | 2024 | Metabolism and energetic balance | 2025 | Active | [61] |
|  | 34 | AR1001 | 2024 | Synaptic plasticity | 2025 | Active | [62] |
|  | 35 | Wujia Yishi | 2025 | Neuroinflammation | 2025 | Active | [63] |
|  | 36 | TRx0237 | 2016 | Tau pathology | 2023 | Innactive/Discontinued/Without updates | [64] |

Abbreviations: disease-modifying immunotherapies (DMI), disease-modifying small molecules (DMSM).
* Years correspond to the respective Alzheimer’s Disease Drug Development Pipeline Reports by Cummings et al.

**References**

[1] Loeffler DA. Intravenous immunoglobulin and Alzheimer's disease: what now? J Neuroinflammation. 2013;10:70.

[2] Novartis. A Study of CAD106 and CNP520 Versus Placebo in Participants at Risk for the Onset of Clinical Symptoms of Alzheimer's Disease (GS1). 2021.

[3] Bateman RJ, Li Y, McDade EM, Llibre-Guerra JJ, Clifford DB, Atri A, et al. Safety and efficacy of long-term gantenerumab treatment in dominantly inherited Alzheimer's disease: an open-label extension of the phase 2/3 multicentre, randomised, double-blind, placebo-controlled platform DIAN-TU trial. Lancet Neurol. 2025;24:316-30.

[4] Doggrell SA. More failure with solanezumab - this time in preclinical Alzheimer's disease. Expert Opin Biol Ther. 2024;24:119-23.

[5] Lozupone M, Dibello V, Sardone R, Castellana F, Zupo R, Lampignano L, et al. Lessons learned from the failure of solanezumab as a prospective treatment strategy for Alzheimer's disease. Expert Opin Drug Discov. 2024;19:639-47.

[6] Seddeik YM, El-Barbari M, Nasim M, Orhan M, Ismail YA. Efficacy of Solanezumab in Early Alzheimer’s Disease: A Systematic Review and Meta Analysis. Alzheimer's & Dementia. 2025;21:e103207.

[7] Padda IS, Parmar M. Aducanumab. StatPearls. Treasure Island (FL)2025.

[8] Biogen. Biogen to Realign Resources for Alzheimer's Disease Franchise. 2024.

[9] NIH. NIA statement on crenezumab trial results: Anti-amyloid drug did not demonstrate a statistically significant clinical benefit in people with inherited form of Alzheimer’s disease. 2022.

[10] Tariot PN, Lopera FS, Rios-Romenets S, Sink KM, Giraldo-Chica M, Acosta-Baena N, et al. Safety and efficacy of crenezumab in cognitively unimpaired carriers of the PSEN1(Glu280Ala) mutation at risk for autosomal-dominant Alzheimer's disease in Colombia (API ADAD Colombia Trial): a phase 2, randomised, double-blind, placebo-controlled trial. Lancet Neurol. 2026;25:147-59.

[11] Perry R, Kipps C, Soto Martin ME, Bozzali M, Logroscino G, Trafford S, et al. Lecanemab for treatment of individuals with early Alzheimer's Disease (AD) who are apolipoprotein E epsilon4 (ApoE epsilon4) non-carriers or heterozygotes. J Prev Alzheimers Dis. 2026;13:100507.

[12] Zimmer JA, Sims JR, Evans CD, Nery ESM, Wang H, Wessels AM, et al. Donanemab in early symptomatic Alzheimer's disease: results from the TRAILBLAZER-ALZ 2 long-term extension. J Prev Alzheimers Dis. 2026;13:100446.

[13] ClinicalTrials.gov. A Study of Remternetug (LY3372993) in Participants With Alzheimer's Disease (TRAILRUNNER-ALZ 1). 2025.

[14] Eisai. EISAI PRESENTS LATEST CLINICAL FINDINGS SUGGESTING INHIBITION OF TAU PROPAGATION BY ANTI-MTBR TAU ANTIBODY E2814 AT THE 17TH CLINICAL TRIALS ON ALZHEIMER'S DISEASE CONFERENCE (CTAD). 2024.

[15] ClinicalTrials.gov. A Study of E2814 With Concurrent Lecanemab Treatment in Participants With Early Alzheimer's Disease. 2026.

[16] Merck. Merck Announces Discontinuation of APECS Study Evaluating Verubecestat (MK-8931) for the Treatment of People with Prodromal Alzheimer’s Disease. 2018.

[17] Decourt B, Noorda K, Noorda K, Shi J, Sabbagh MN. Review of Advanced Drug Trials Focusing on the Reduction of Brain Beta-Amyloid to Prevent and Treat Dementia. J Exp Pharmacol. 2022;14:331-52.

[18] ClinicalTrials.gov. A Study of Sodium Oligomannate (GV-971) in Participants With Mild to Moderate Alzheimer's Disease (GREEN MEMORY). 2022.

[19] ClinicalTrials.gov. A Study of CAD106 and CNP520 Versus Placebo in Participants at Risk for the Onset of Clinical Symptoms of Alzheimer's Disease (GS1). 2015. p. July 8, 2021.

[20] Lozupone M, Berardino G, Mollica A, Sardone R, Dibello V, Zupo R, et al. ALZT-OP1: an experimental combination regimen for the treatment of Alzheimer's disease. Expert Opin Investig Drugs. 2022;31:759-71.

[21] Lawlor B, Segurado R, Kennelly S, Olde Rikkert MGM, Howard R, Pasquier F, et al. Nilvadipine in mild to moderate Alzheimer disease: A randomised controlled trial. PLoS Med. 2018;15:e1002660.

[22] Wessels AM, Tariot PN, Zimmer JA, Selzler KJ, Bragg SM, Andersen SW, et al. Efficacy and Safety of Lanabecestat for Treatment of Early and Mild Alzheimer Disease: The AMARANTH and DAYBREAK-ALZ Randomized Clinical Trials. JAMA Neurol. 2020;77:199-209.

[23] Burstein AH, Sabbagh M, Andrews R, Valcarce C, Dunn I, Altstiel L. Development of Azeliragon, an Oral Small Molecule Antagonist of the Receptor for Advanced Glycation Endproducts, for the Potential Slowing of Loss of Cognition in Mild Alzheimer's Disease. J Prev Alzheimers Dis. 2018;5:149-54.

[24] ClinicalTrials.gov. Evaluation of the Efficacy and Safety of Azeliragon (TTP488) in Patients With Mild Alzheimer's Disease (STEADFAST). 2015.

[25] ClinicalTrials.gov. 2-Year Extension Study of Azeliragon in Subjects With Alzheimer's Disease (STEADFAST Extension). 2016.

[26] Novak G, Streffer JR, Timmers M, Henley D, Brashear HR, Bogert J, et al. Long-term safety and tolerability of atabecestat (JNJ-54861911), an oral BACE1 inhibitor, in early Alzheimer's disease spectrum patients: a randomized, double-blind, placebo-controlled study and a two-period extension study. Alzheimers Res Ther. 2020;12:58.

[27] Sperling R, Henley D, Aisen PS, Raman R, Donohue MC, Ernstrom K, et al. Findings of Efficacy, Safety, and Biomarker Outcomes of Atabecestat in Preclinical Alzheimer Disease: A Truncated Randomized Phase 2b/3 Clinical Trial. JAMA Neurol. 2021;78:293-301.

[28] Burns DK, Alexander RC, Welsh-Bohmer KA, Culp M, Chiang C, O'Neil J, et al. Safety and efficacy of pioglitazone for the delay of cognitive impairment in people at risk of Alzheimer's disease (TOMMORROW): a prognostic biomarker study and a phase 3, randomised, double-blind, placebo-controlled trial. Lancet Neurol. 2021;20:537-47.

[29] Lin HC, Chung CH, Chen LC, Wang JY, Chen CC, Huang KY, et al. Pioglitazone use increases risk of Alzheimer's disease in patients with type 2 diabetes receiving insulin. Sci Rep. 2023;13:6625.

[30] Craft S, Raman R, Chow TW, Rafii MS, Sun CK, Rissman RA, et al. Safety, Efficacy, and Feasibility of Intranasal Insulin for the Treatment of Mild Cognitive Impairment and Alzheimer Disease Dementia: A Randomized Clinical Trial. JAMA Neurol. 2020;77:1099-109.

[31] Dubois B, Lopez-Arrieta J, Lipschitz S, Doskas T, Spiru L, Moroz S, et al. Masitinib for mild-to-moderate Alzheimer's disease: results from a randomized, placebo-controlled, phase 3, clinical trial. Alzheimers Res Ther. 2023;15:39.

[32] ClinicalTrials.gov. A 24-Month Study to Evaluate the Efficacy and Safety of Elenbecestat (E2609) in Participants With Early Alzheimer's Disease (MissionAD1). 2016.

[33] ClinicalTrials.gov. Brain Amyloid and Vascular Effects of Eicosapentaenoic Acid (BRAVE-EPA). 2017.

[34] Macfarlane S, Grimmer T, Teo K, O'Brien TJ, Woodward M, Grunfeld J, et al. Blarcamesine for the treatment of Early Alzheimer's Disease: Results from the ANAVEX2-73-AD-004 Phase IIB/III trial. J Prev Alzheimers Dis. 2025;12:100016.

[35] Sabbagh MN, Decourt B. COR388 (atuzaginstat): an investigational gingipain inhibitor for the treatment of Alzheimer disease. Expert Opin Investig Drugs. 2022;31:987-93.

[36] ClinicalTrials.gov. GAIN Trial: Phase 2/​3 Study of COR388 in Subjects With Alzheimer's Disease. 2019.

[37] ClinicalTrials.gov. Risk Reduction for Alzheimer's Disease (rrAD). 2017.

[38] Szabo-Reed AN, Vidoni E, Binder EF, Burns J, Cullum CM, Gahan WP, et al. Rationale and methods for a multicenter clinical trial assessing exercise and intensive vascular risk reduction in preventing dementia (rrAD Study). Contemp Clin Trials. 2019;79:44-54.

[39] Sen A, Toniolo S, Tai XY, Akinola M, Symmonds M, Mura S, et al. Safety, tolerability, and efficacy outcomes of the Investigation of Levetiracetam in Alzheimer's disease (ILiAD) study: a pilot, double-blind placebo-controlled crossover trial. Epilepsia Open. 2024;9:2353-64.

[40] Bakker A, Rani N, Mohs R, Gallagher M. The HOPE4MCI study: AGB101 treatment slows progression of entorhinal cortex atrophy in APOE epsilon4 non-carriers with mild cognitive impairment due to Alzheimer's disease. Alzheimers Dement (N Y). 2024;10:e70004.

[41] Mohs R, Bakker A, Rosenzweig-Lipson S, Rosenblum M, Barton RL, Albert MS, et al. The HOPE4MCI study: A randomized double-blind assessment of AGB101 for the treatment of MCI due to AD. Alzheimers Dement (N Y). 2024;10:e12446.

[42] PRNewswire. Biohaven Provides Update On Phase 2/3 Trial And Alzheimer's Disease Program. 2021.

[43] Luchsinger JA, Devanand D, Goldberg TE, Cammack S, Hernandez-Santiago G, Oishi K, et al. Protocol for a Randomized Phase II/III Double-Blind Placebo-Controlled Trial to Evaluate the Safety and Efficacy of Extended-Release Metformin in Amnestic Mild Cognitive Impairment: Metformin in Alzheimer Dementia Prevention (MAP). Alzheimer Dis Assoc Disord. 2025;39:123-33.

[44] ClinicalTrials.gov. A Phase III Multi Regional Clinical Trial (MRCT) of Tricaprilin in Mild to Moderately Severe Probable Alzheimer's Disease With Optional Open Label Extension. 2022.

[45] ClinicalTrials.gov. Tricaprilin Phase 3 ALTER-AD (Alternative-Alzheimer Disease) Study (ALTER-AD). 2023.

[46] Reading CL, Ahlem CN, Murphy MF. NM101 Phase III study of NE3107 in Alzheimer's disease: rationale, design and therapeutic modulation of neuroinflammation and insulin resistance. Neurodegener Dis Manag. 2021;11:289-98.

[47] Reading CL, Yan J, Testa MA, Simonson DC, Javaid H, Schmunk L, et al. An exploratory analysis of bezisterim treatment associated with decreased biological age acceleration, and improved clinical measure and biomarker changes in mild-to-moderate probable Alzheimer's disease. Front Neurosci. 2025;19:1516746.

[48] ClinicalTrials.gov. Prevention of Cognitive Decline in Older Adults With Low Dha/​Epa Index in Red Blood Cells (LO-MAPT). 2018.

[49] Mirzaei M, Ahmadi N, Bagheri Fahraji B, Ardekani AM, Rahimdel A, Soltani MH, et al. A randomized clinical trial evaluating Hydralazine's efficacy in early-stage Alzheimer's disease: The EHSAN Study. Sci Rep. 2024;14:28837.

[50] ClinicalTrials.gov. Effect of Hydralazine on Alzheimer's Disease (EHSAN). 2021.

[51] ClinicalTrials.gov. Evaluating the Efficacy and Safety of Nilotinib BE in Subjects With Early Alzheimer's Disease (NILEAD). 2021.

[52] ClinicalTrials.gov. A Research Study Investigating Semaglutide in People With Early Alzheimer's Disease (EVOKE) (EVOKE). 2021.

[53] ClinicalTrials.gov. A Research Study Investigating Semaglutide in People With Early Alzheimer's Disease (EVOKE Plus) (EVOKE Plus). 2021.

[54] Kupiec JW, Porsteinsson AP, Turner RS, Hendrix S, Mallinckrodt C, Khan A, et al. Phase 3 randomized clinical trials of simufilam in mild-to-moderate Alzheimer's disease. J Prev Alzheimers Dis. 2026:100469.

[55] Abushakra S, Porsteinsson AP, Sabbagh M, Watson D, Power A, Liang E, et al. APOLLOE4 Phase 3 study of oral ALZ-801/valiltramiprosate in APOE epsilon4/epsilon4 homozygotes with early Alzheimer's disease: Trial design and baseline characteristics. Alzheimers Dement (N Y). 2024;10:e12498.

[56] Abushakra S, Power A, Watson D, Porsteinsson A, Sabbagh M, MacSweeney E, et al. Clinical Efficacy, Safety and Imaging Effects of Oral Valiltramiprosate in APOEepsilon4/epsilon4 Homozygotes with Early Alzheimer's Disease: Results of the Phase III, Randomized, Double-Blind, Placebo-Controlled, 78-Week APOLLOE4 Trial. Drugs. 2025;85:1455-72.

[57] Porsteinsson AP, Sabbagh M, Tariot PN, Church KJ, San Martin J, Ooi KC, et al. Fosgonimeton in mild-to-moderate Alzheimer's disease. J Alzheimers Dis Rep. 2025;9:25424823251405817.

[58] ClinicalTrials.gov. Piromelatine 20 mg in Participants With Mild Dementia Due to Alzheimer's Disease. 2022.

[59] ClinicalTrials.gov. GV1001 Subcutaneous for the Treatment of Moderate to Severe Alzheimer's Disease(AD). 2022.

[60] ClinicalTrials.gov. A Double-blind Dual Study Assessing Safety and Efficacy of Buntanetap in Participants With Early AD. 2024.

[61] ClinicalTrials.gov. GnRH Therapy on Cognition in Down Syndrome. 2020.

[62] ClinicalTrials.gov. Phase 3, Double-blind, Randomized, Placebo-controlled Trial to Evaluate the Efficacy and Safety of AR1001 in Participants With Early Alzheimer's Disease (Polaris-AD). 2022.

[63] ClinicalTrials.gov. Phase 3 Clinical Trial of Wujia Yizhi Granules in the Treatment of Mild-to-moderate Alzheimer's Dementia (Syndrome of Deficiency of Spleen and Kidney). 2024.

[64] Gauthier S, Feldman HH, Schneider LS, Wilcock GK, Frisoni GB, Hardlund JH, et al. Efficacy and safety of tau-aggregation inhibitor therapy in patients with mild or moderate Alzheimer's disease: a randomised, controlled, double-blind, parallel-arm, phase 3 trial. Lancet. 2016;388:2873-84.

**Supplementary Table 2. Chronological distribution of Alzheimer’s disease drug candidates entering Phase III clinical trials**

| **Year*** | **DMI**** | **DMSM**** | **Total** | **%DMI** | **%DMSM** | **%Global** |
| --- | --- | --- | --- | --- | --- | --- |
| **2016** | 5 | 12 | 17 | 10.6 | 25.5 | 36.2 |
| **2017** | 1 | 1 | 2 | 2.1 | 2.1 | 4.3 |
| **2018** | 0 | 1 | 1 | 0.0 | 2.1 | 2.1 |
| **2019** | 0 | 5 | 5 | 0.0 | 10.6 | 10.6 |
| **2020** | 1 | 2 | 3 | 2.1 | 4.3 | 6.4 |
| **2021** | 0 | 2 | 2 | 0.0 | 4.3 | 4.3 |
| **2022** | 1 | 5 | 6 | 2.1 | 10.6 | 12.8 |
| **2023** | 2 | 3 | 5 | 4.3 | 6.4 | 10.6 |
| **2024** | 0 | 4 | 4 | 0.0 | 8.5 | 8.5 |
| **2025** | 1 | 1 | 2 | 2.1 | 2.1 | 4.3 |
| **Total** | **11** | **36** | **47** | **23.4** | **76.6** | **100.0** |

Abbreviations: disease-modifying immunotherapies (DMI), disease-modifying small molecules (DMSM).
* Years correspond to the respective Alzheimer’s Disease Drug Development Pipeline Reports by Cummings et al.
** Number of DMI or DMSM reported as entering Phase III trial

**Supplementary Table 3. Targets of Alzheimer’s disease drug candidates entering Phase III clinical trials**

| **Target** | **#Drug candidates** | **%** |
| --- | --- | --- |
| **Amyloid pathology** | 21 | 44.7 |
| **Synaptic plasticity** | 9 | 19.1 |
| **Neuroprotection** | 8 | 17.0 |
| **Neuroinflammation** | 7 | 14.9 |
| **Metabolism and Energetic Balance** | 5 | 10.6 |
| **Oxidative stress** | 4 | 8.5 |
| **Tau pathology** | 3 | 6.4 |
| **Other pathways** | 3 | 6.4 |
| **Neurogenesis** | 2 | 4.3 |
| **Vasculature** | 1 | 2.1 |
|  |  |  |
| **Multitarget** | 12 | 25.5 |
